# Effectiveness of Inactivated Influenza Vaccines in Preventing Influenza-Associated Deaths and Hospitalizations among Ontario Residents Aged ≥65 Years: Estimates with Generalized Linear Models Accounting for Healthy Vaccinee Effects

**DOI:** 10.1371/journal.pone.0076318

**Published:** 2013-10-16

**Authors:** Benjamin J. Ridenhour, Michael A. Campitelli, Jeffrey C. Kwong, Laura C. Rosella, Ben G. Armstrong, Punam Mangtani, Andrew J. Calzavara, David K. Shay

**Affiliations:** 1 Influenza Division, United States Centers for Disease Control and Prevention, Atlanta, Georgia, United States of America; 2 Eck Institute for Global Health, Department of Biological Sciences, University of Notre Dame, South Bend, Indiana, United States of America; 3 Institute for Clinical Evaluative Sciences, Toronto, Ontario, Canada; 4 Ontario Agency for Health Protection and Promotion, Toronto, Ontario, Canada; 5 Dalla Lana School of Public Health, University of Toronto, Toronto, Ontario, Canada; 6 Department of Family and Community Medicine, University of Toronto, Toronto, Ontario, Canada; 7 Department of Social and Environmental Health Research, London School of Hygiene and Tropical Medicine, London, United Kingdom; 8 Department of Epidemiology and Population Health, London School of Hygiene and Tropical Medicine, London, United Kingdom; Centers for Disease Control and Prevention, United States of America

## Abstract

**Background:**

Estimates of the effectiveness of influenza vaccines in older adults may be biased because of difficulties identifying and adjusting for confounders of the vaccine-outcome association. We estimated vaccine effectiveness for prevention of serious influenza complications among older persons by using methods to account for underlying differences in risk for these complications.

**Methods:**

We conducted a retrospective cohort study among Ontario residents aged ≥65 years from September 1993 through September 2008. We linked weekly vaccination, hospitalization, and death records for 1.4 million community-dwelling persons aged ≥65 years. Vaccine effectiveness was estimated by comparing ratios of outcome rates during weeks of high versus low influenza activity (defined by viral surveillance data) among vaccinated and unvaccinated subjects by using log-linear regression models that accounted for temperature and time trends with natural spline functions. Effectiveness was estimated for three influenza-associated outcomes: all-cause deaths, deaths occurring within 30 days of pneumonia/influenza hospitalizations, and pneumonia/influenza hospitalizations.

**Results:**

During weeks when 5% of respiratory specimens tested positive for influenza A, vaccine effectiveness among persons aged ≥65 years was 22% (95% confidence interval [CI], −6%–42%) for all influenza-associated deaths, 25% (95% CI, 13%–37%) for deaths occurring within 30 days after an influenza-associated pneumonia/influenza hospitalization, and 19% (95% CI, 4%–31%) for influenza-associated pneumonia/influenza hospitalizations. Because small proportions of deaths, deaths after pneumonia/influenza hospitalizations, and pneumonia/influenza hospitalizations were associated with influenza virus circulation, we estimated that vaccination prevented 1.6%, 4.8%, and 4.1% of these outcomes, respectively.

**Conclusions:**

By using confounding-reducing techniques with 15 years of provincial-level data including vaccination and health outcomes, we estimated that influenza vaccination prevented ∼4% of influenza-associated hospitalizations and deaths occurring after hospitalizations among older adults in Ontario.

## Introduction

Influenza viruses are associated with substantial morbidity annually, and persons aged ≥65 years are among those at highest risk of serious outcomes following influenza infection [1]–[3]. Annual influenza vaccination is recommended for older adults in Canada, the United States, and many other developed countries [4], [5]. However, the effectiveness of vaccination among older adults is a subject of considerable debate. The only large randomized placebo-controlled clinical trial of inactivated influenza vaccine in adults aged ≥60 years was conducted during a single season; efficacy was 58% for prevention of serologically-confirmed influenza in symptomatic subjects [6], [7]. All other large studies of influenza vaccine effects among older persons have used observational data, typically from retrospective cohort studies. For example, retrospective cohort studies conducted with US health plan data reported that influenza vaccine effectiveness (VE) in community-dwelling elderly persons was 47% for preventing all-cause mortality and 27% for preventing pneumonia/influenza hospitalizations [8], [9].

Of course, the results from any observational study are susceptible to bias [8]–[10]. It has been suggested that VE estimates from cohort studies using electronic health records were susceptible specifically to “healthy vaccinee” bias [10]–[12], and previous work has shown that statistical adjustment for covariates available in electronic health records, including ICD-9-CM-based diagnostic codes, do not control for this bias [13]. However, there is no alternative to using observational data for assessing vaccine effects against serious outcomes of influenza infections, given the rarity of these outcomes. Clearly, methods more advanced than those commonly used in “standard” cohort studies are needed to control for unmeasured confounders in vaccine studies conducted among older adults.

Observational studies of vaccine effects are plagued not only by the potential for confounding, but also by the non-specific nature of the outcomes commonly used in these studies, such as community-acquired pneumonia. A recent simulation study demonstrated that if an influenza vaccine had a true VE against influenza infection of 55%, attaining vaccine coverage of 38% in a population would lead to a VE against pneumonia of just 7% (95% confidence interval [CI] 0%–25%), given assumptions about attack rates and risk of pneumonia following influenza infection based on recent data [14]. Most VE studies conducted among older persons have estimated effectiveness by comparing rates of serious, but not influenza-specific, outcomes among vaccinated and unvaccinated persons during weeks when influenza viruses circulated, with adjustment for potential confounders. However, even during the discrete influenza seasons found in temperate regions, most of these outcomes (e.g., all ICD-9-CM-coded pneumonia/influenza hospitalizations) are not associated with influenza infections. For example, among adults hospitalized with lower respiratory tract infections during winter seasons, only 4%–20% have evidence of influenza infection [15]–[18]. Thus, use of a more specific outcome than pneumonia, for example, could improve the precision of influenza VE estimates, and perhaps decrease the likelihood of confounding as well. It is well known that influenza epidemics are associated with increases in pneumonia/influenza hospitalizations and deaths above expected, smoothed seasonal baselines [3], [15]. We propose that using these ‘excess’ or influenza-associated outcomes in observational cohort studies of influenza VE, rather than all the outcomes occurring while influenza is circulating, could lead to more precise and less-biased VE estimates.

Some studies have sought to reduce the likelihood of bias by comparing adjusted VE for non-specific outcomes during weeks when influenza is circulating and weeks when it is not. For example, a UK study observed VE against respiratory hospitalizations of 21% (95% CI, 17%–26%) and against respiratory-coded deaths of 12% (95% CI, 8%–16%) during weeks when influenza circulated, but not in weeks when it did not [16]. Jackson *et al*. conducted a case-control study estimating VE for preventing community-acquired pneumonia. They reviewed individual medical records to obtain information on possible confounders not contained in traditional electronic health records (e.g., smoking history, frailty, severity of lung and heart disease) and sought to identify a set of covariates that resulted in a null VE during the pre-influenza season period in their US health plan population, and used the same covariates to estimate VE during the influenza season [17]. The estimated VE in this study was 8% (95% CI, −10%–23%), very similar to the estimate of 7% (95% CI, 0%–12%) found in the simulation study for VE against a non-specific pneumonia, given a VE against influenza infection of 55% [14].

Other studies have used novel statistical approaches in an attempt to calculate less-biased VE estimates. For example, Armstrong *et al*. estimated VE by comparing mortality rate ratios among vaccinated and unvaccinated subjects as influenza activity increased, thus adjusting for baseline differences between these two groups that affect mortality risk [18], [19]. In that study, when influenza virus circulation was at its peak, defined as at or above the 90th percentile of laboratory detections for influenza, VE for the prevention of influenza-associated deaths was 85% (95% CI, 13%–100%). As mortality is a rare outcome even in elderly subjects and this method is data-intensive, the confidence limits covered the entire meaningful range, even though the study included ∼25,000 subjects. Fireman *et al*. sought to reduce healthy vaccinee bias by estimating influenza VE with another novel method they described as a “case-centered logistic regression.” Their analysis was similar conceptually to a finely stratified case-cohort study, with the expected odds of vaccination in the underlying population stratified by age, sex, and day, and compared with the actual odds of vaccination in cases in the same strata [20]. Fireman estimated VE to be 4.6% (95% CI, 0.7%–8.3%) for preventing all deaths during influenza seasons. Based on the authors' calculations, this VE for prevention of all deaths implied a VE of 47% against influenza-associated deaths. This study used data from a single US managed heath care plan, and thus the ability to generalize its findings to broader populations is unknown.

In this study, we sought to provide robust and generalizable estimates of influenza VE by using confounding-reducing methods with the entire community-dwelling population of Ontario aged ≥65 years during 15 influenza seasons. Our outcome measures represent influenza-associated events, rather than all events occurring during defined periods of influenza circulation.

## Methods

### Design, setting, and participants

We conducted a population-based retrospective cohort study using Ontario data from 1993–1994 through 2007–2008. At each season's index date (the Sunday before 1 September), a study population was established with Ontario residents aged ≥65 years who were eligible to receive universal, publicly insured health care services. These subjects had free access to hospital care, physician services, and influenza vaccines. The study cohort was restricted to non-institutionalized persons who had been in contact with the health care system within 3 years of the index date, to exclude individuals who may have moved, resided in the province rarely, or died but had not yet been classified as deceased in provincial records.

We linked administrative health datasets for each study subject by using encrypted health card numbers as unique identifiers. Only de-identified, aggregated data were used for data analyses.

### Ethics Statement

Ethics approval was obtained from the Research Ethics Board of Sunnybrook Health Sciences Centre, Toronto, Canada. This study used routinely collected health information from the province of Ontario that was aggregated into weekly counts. The mortality, hospitalization and physician services data used contained no personal identifiers. The use of aggregate data and data without personal identifiers precluded the need to obtain informed consent. Additionally, the Institute of Clinical Evaluative Sciences (ICES) is named as a prescribed entity under section 45 of the *Personal Health Information Protection Act* (Ontario Regulation 329/04, Section 18). Under this designation, ICES can receive and use health information without consent for purposes of analysis and compiling statistical information about the health care system of Ontario.

### Study population

The study included all community-dwelling individuals aged ≥65 years in Ontario from 1993 through 2008 who met the inclusion criteria (Table 1).

**Table 1:** Number of individuals included in the study, influenza vaccinations, and outcomes for each study year.

				All-cause deaths		30-day pneumonia/influenza deaths		Pneumonia/influenza hospitalizations	
Study Year	Percentage A/H3N2 (%)	N	Influenza vaccinations (%)	Vaccinated	Unvaccinated	Vaccinated	Unvaccinated	Vaccinated	Unvaccinated
1993/1994	99	1,214,078	437,520 (36.0)	12,414	33,265	1,187	2,644	6,296	13,071
1994/1995	80	1,247,657	471,083 (37.8)	13,157	33,765	1,307	2,791	6,948	13,801
1995/1996	8	1,279,540	505,867 (39.5)	14,244	33,601	1,334	2,745	6,903	12,974
1996/1997	78	1,308,165	561,566 (42.9)	15,812	32,317	1,635	2,922	8,369	13,387
1997/1998	73	1,334,162	609,439 (45.7)	16,614	31,624	1,817	2,975	9,534	13,169
1998/1999	72	1,359,914	635,658 (46.7)	17,215	31,043	1,938	2,938	9,995	13,835
1999/2000	93	1,382,719	699,266 (50.6)	18,547	29,649	2,128	2,891	11,184	14,724
2000/2001	1	1,408,542	834,607 (59.3)	20,853	27,340	2,124	2,386	11,740	11,846
2001/2002	64	1,434,909	837,831 (58.4)	20,534	27,792	2,174	2,538	12,015	12,585
2002/2003	2	1,461,549	814,275 (55.7)	19,728	29,223	1,967	2,517	10,103	11,989
2003/2004	99	1,488,524	880,014 (59.1)	20,628	28,427	2,190	2,639	11,788	12,293
2004/2005	75	1,516,411	882,334 (58.2)	20,883	28,400	2,156	2,573	12,184	12,441
2005/2006	39	1,543,667	878,595 (56.9)	19,432	28,538	1,908	2,396	10,313	11,689
2006/2007	91	1,580,883	846,844 (53.6)	17,976	31,918	1,840	2,871	9,749	13,895
2007/2008	24	1,620,199	825,243 (50.9)	17,941	33,163	1,836	2,920	9,849	13,919
Average	60	1,412,061	714,676 (50.6)	17,731	30,671	1,836	2,716	9,798	13,041

### Influenza vaccination status

Vaccination status was ascertained from influenza-specific and generic vaccination codes for physician billing claims submitted to the Ontario Health Insurance Plan, a universal insurance plan which covers all residents of Ontario. This database contains claims for outpatient visits from approximately 98% of Ontario physicians [21]. Because influenza-specific vaccination codes were introduced in 1998, and their use gradually increased after introduction, we also used generic vaccination codes billed during weeks when the total number of these claims exceeded a summer baseline rate (typically between late September and late December). Although these generic vaccination claims included those for other vaccines, based on previous analyses and our study data, ∼96% represented influenza vaccines (Figure S1) [22]. Compared with self-reported influenza vaccination, the combination of influenza-specific and generic vaccination codes had sensitivity of 75%, specificity of 90%, positive predictive value of 96%, and negative predictive value of 54% among adults aged ≥65 years [23]. Cohort members were classified as unvaccinated at each season's index date; we defined an individual as “immunized” two weeks after the billing claim service date to account for the delay from vaccination to development of specific humoral immunity to vaccine strains [24].

### Outcomes

Ontario's Registered Persons Database has key demographic information about each person who has ever received an Ontario health card [25]. It was used to ascertain vital status and date of death, if deceased, for individuals in the cohort. Specific cause of death information was not available for this analysis, so we used as outcomes all-cause mortality and mortality within 30 days of a pneumonia/influenza hospitalization (see below) to provide a more specific outcome for deaths in which pneumonia/influenza likely played a role (hereafter referred to as ‘30-day pneumonia/influenza death’) [26].

Hospitalizations for pneumonia/influenza (ICD-9-CM codes 480-487; ICD-10-CM codes J10-J18) were ascertained from the Canadian Institute of Health Information's Discharge Abstract Database; this database contains detailed information on diagnoses and procedures for admissions to all acute-care hospitals in Canada [27]. We included hospitalizations for which any of the listed codes were found in the discharge abstract.

To confirm the specificity of the VE estimates for pneumonia/influenza hospitalizations, we also examined hospitalizations for urinary tract infections (ICD-9-CM codes 590, 595, 599.0; ICD-10-CM codes N10, N12, N13.6, N15.1, N30, N39.0) as a ‘negative control’ outcome for which influenza vaccination is not expected to have an effect [28].

### Influenza surveillance

We obtained weekly influenza viral surveillance data from a provincial network of sentinel laboratories that submit weekly reports of numbers of tests performed (using predominantly viral culture or direct antigen detection methods) and numbers of positive tests for influenza A and B to the Public Health Agency of Canada. For each virus, we used the weekly percentage of tests positive as our measure of viral circulation.

### Temperature

Rates of serious illness and mortality are associated with fluctuations in temperature [29], as is influenza activity in temperate regions. Thus, we sought consistently collected temperature data to adjust for seasonal temperature variations. Daily measures of mean, maximum, and minimum temperature at the weather station located at Lester B. Pearson International airport in Toronto were obtained from the Ontario Climate Centre. Seventy-five percent of Ontario's population resides within 150 kilometers of this station [30]. Because we sought to adjust for weekly associations between temperature and mortality — rather than estimate potentially causal daily associations between temperature and mortality — we concluded that data from this station was sufficient to control for the possible confounding effects of temperature trends in Ontario on VE.

### Vaccine effectiveness calculation

We used Poisson models to regress weekly outcomes against the weekly proportion of influenza tests that were positive for influenza A or B. Similar methods have been used in time-series analyses of air pollution and mortality [31]. Our statistical approach was similar to the methods described by Armstrong, *et al* [18] for estimating VE against influenza-associated events by using log-linear generalized linear models. VE was modeled as the ratio in outcome rates during periods of varying influenza activity among vaccinated and unvaccinated cohort members. Because the vaccinated population was compared with the vaccinated population at previous time points, and likewise for the unvaccinated population, this approach adjusted implicitly for baseline health status indicators like mobility, frailty, and dementia.

Vaccine effectiveness was represented as: VE =  (RR(u) − RR(v))/(RR(u) − 1) = 1 − (1 − RR(v))/(1 − RR(u)) where RR was the incidence of an outcome during an influenza period divided by the incidence outside an influenza period among the vaccinated (v) and unvaccinated (u) groups [19]. The estimator for VE can be expressed by using terms from a generalized linear model as VE = (1 − exp(β_x*v_ x))/(1 − exp(−*β_x_ x*)) where *β_x_* was the estimated regression coefficient for the influenza circulation variable (i.e., the proportion of specimens testing positive for influenza A or B), and β_x*v_ was the estimated regression coefficient for the influenza circulation multiplied by vaccination status variable (i.e., the interaction term between vaccination status and influenza circulation). Note that our estimate of VE is actually a continuous function that depends on the level of influenza circulation. We present results for hypothetical weeks when 5% or 10% of tests submitted were positive for influenza viruses, and defined these weeks as those with moderate and high levels of influenza circulation, respectively.

### Regression models

We regressed each of the outcomes each week against respiratory specimens that tested positive for influenza. The ratios in outcome rates during periods of varying influenza activity in vaccinated and unvaccinated individuals were then compared. Outcome rates were estimated using log-linear regression models. Because the data were found to be overdispersed, we used a quasi-poisson error distribution. An offset term was included to account for the appropriate population denominator for each of the outcome variables. The model can be stated as *y*∼Quasi-poisson(μ, θ); μ = *N* exp (Xβ+ε) where *y* is the outcome, log(*N*) is the offset, Xβ is the linear predictor, and the error term ε is distributed to allow for overdispersion θ. The offset used in our models was the logarithm of the number of person-days of observation in a particular stratum for a given week. All analyses were performed at the weekly level.

Our modeling strategy was as follows. First, a ‘baseline’ model was constructed that consisted of nothing but an intercept and the offset term, i.e.

Outcome  =  Intercept + log(Exposure)

and each of our outcomes was modeled separately. Additional terms were added via forward selection to our baseline model using analysis of deviance. Upon inclusion into the model, terms could be subsequently dropped via backwards elimination. Alternating forward and reverse selection steps were performed until either no more potential variables were available or no statistically significant (p<0.05) additions could be made.

Potential variables for inclusion into models included: vaccine status (vaccinated/unvaccinated), influenza A circulation, influenza B circulation, sex (male, female), age group (ages 65–74 years or ≥75 years), week of the year (i.e., 1…52), week of the study period (i.e., 1…783), weekly mean temperature, and a dichotomous variable to account for introduction of Ontario's Universal Influenza Immunization Program (UIIP) on 1 September 2000. After UIIP began, the entire population of Ontario aged ≥6 months, regardless of age or underlying medical condition, became eligible to receive free seasonal influenza vaccination. Main effects of any variable had to be present before interaction effects could enter the model. Only interactions with vaccine status were considered. For example, a vaccine status and age group interaction could be included in the model once the main effects of vaccine status and age group had been included. Inclusion of any vaccine status and time variables in our model indicated the presence of time-varying biases of vaccination status and allowed us to control for these effects appropriately. Vaccine effectiveness was calculated only if a vaccine status and influenza circulation interaction effect was included in a regression model.

Natural cubic spline functions were used to model the association of three covariates on influenza-associated events: week of the year, week of the study, and weekly mean temperature. Natural splines are cubic functions “tied” together at *n* “knots” and have *n +*1 degrees of freedom (i.e., if d.f.  =  1 then there are no knots, and the spline is simply a cubic function). Splines for these three variables with 1 d.f. to 6 d.f. were evaluated for possible inclusion into our models. Once a spline for a given variable entered a statistical model, no other spline for that variable could enter the model. In other words, if a spline for mean temperature with 2 d.f. was used based on analysis of deviance, then splines for mean temperature with 1, 3, 4, 5, and 6 d.f. were not considered. If the spline with 2 d.f. was subsequently dropped, then any of the spline representations of temperature could be evaluated for inclusion again. The use of natural splines for mean temperature and week of the year allowed the model to control for the strong seasonality of our outcomes in a more logical and flexible manner than the sine/cosine harmomic variables often used in studies seeking to identify influenza-associated outcomes [1]–[3], [32]. Similarly, using a natural spline to represent calendar week in models was more flexible in capturing long-term time trends in outcomes than simple polynomials.

A list of the predictors included in the final models (Table S1) and an enumeration of those predictors from the model of each outcome (Table S2) are provided in the Supplemental Materials. Because of autocorrelation present in our time-series, we applied Newey-West estimators to the parameter variance-covariance matrix to correct for the observed autocorrelation and thereby provide consistent estimates of the regression parameters [33].

### Estimates of cases averted and numbers needed to vaccinate

We estimated the absolute number of outcomes averted by influenza vaccination in Ontario by using the predicted number of cases (*ŷ*) in the vaccinated group and the vaccine effect on the estimate of excess cases while influenza virus circulation varied (β_x*v_). By setting the effect of vaccination to zero (β_x*v_ = 0), we estimated the number of cases (*y′*) that would have occurred in the vaccinated population if vaccine had no effect or if it was never delivered. The number of cases averted was defined as (*y′ – ŷ*). We calculated the proportion of outcomes prevented in the vaccinated group (i.e., the proportion averted among vaccinees) by dividing the number of cases averted by the total number of cases in the vaccinated group during weeks of influenza virus circulation with the number of cases averted added back into the denominator. This proportion can be interpreted as an estimate of VE against all occurrences of these outcomes during periods of influenza virus circulation. Furthermore, for each outcome we calculated the number needed to vaccinate (NNV) to prevent one outcome by dividing the mean annual number of influenza vaccines administered by the mean annual number of cases averted per season.

All analyses were conducted using R software (R Development Core Team, R Foundation for Statistical Computing, Vienna, Austria. http://www.R-project.org.).

## Results

**Figure 1:**
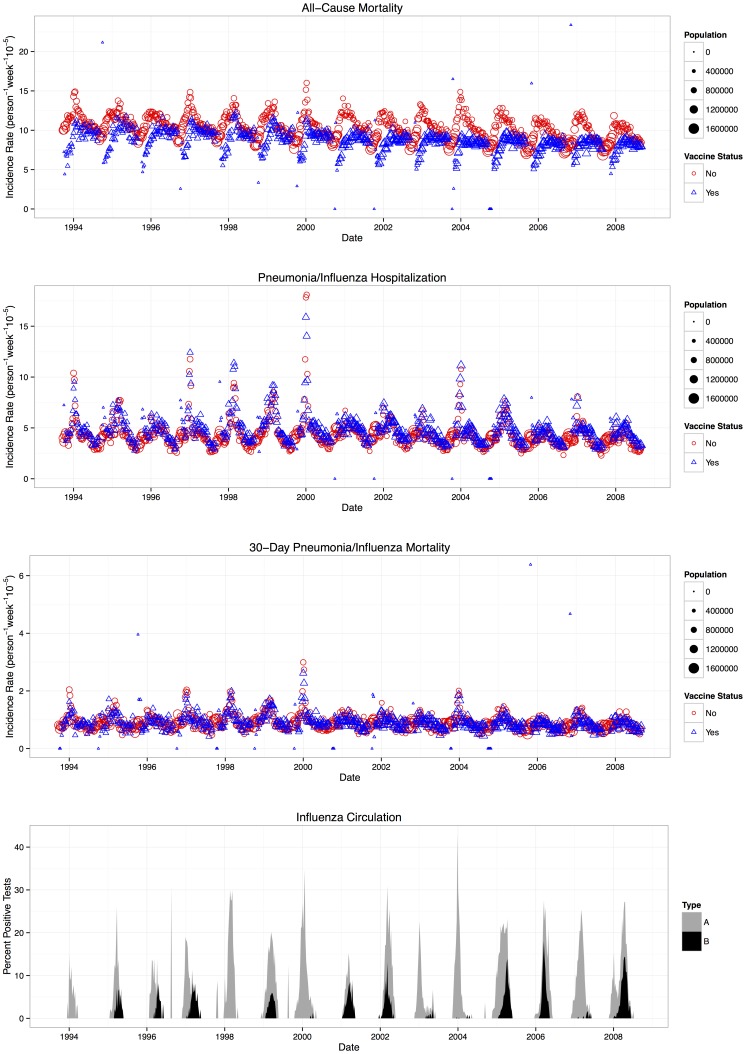
Weekly trends of influenza viral surveillance outcome rates among individuals aged ≥65 years. In the top three panels, weekly outcome rates are indicated by red symbols for unvaccinated individuals and blue symbols for vaccinated individuals; the size of the symbol reflects the number of individuals in a category. The panels show all-cause mortality, 30-day pneumonia/influenza mortality, and pneumonia/influenza hospitalization from top to bottom, respectively. In the bottom panel, weekly percentages of specimens testing positive for either influenza A or B are represented by gray and black bars (respectively). The sum of the black bar and gray bar shows the total percent positive for the week (i.e., the data for influenza A and B are *not* overlaid).

During the 15 study seasons, an annual average of 48,402 all-cause deaths, 4,552 30-day pneumonia/influenza deaths, and 22,839 pneumonia/influenza hospitalizations occurred among community-dwelling adults aged ≥65 years in Ontario (Table 1; outcomes stratified by age group in Table S3). During this period, 334,797 influenza tests were performed, with 26,753 positive for influenza A viruses and 6,774 positive for influenza B viruses (8% and 2% positive, respectively). Influenza viruses were detected during 479 weeks (61% of study weeks). Greater than or equal to 5% or ≥10% of tests were positive for influenza during 218 weeks (28%) and 132 weeks (17%), respectively. During each 52-week period, ≥5% and ≥10% of tests were positive for influenza during a median of 14 and 10 weeks, respectively. Each of the outcomes demonstrated weekly seasonality, with spikes coinciding with periods of influenza activity among both vaccinated and unvaccinated individuals (Figure 1).

In the statistical models for each of the outcomes we examined, the presence of a vaccine status and influenza circulation interaction variable (determined by using analysis of deviance) indicated evidence of an effect of vaccination on influenza-associated events. When we used hospitalizations for UTIs as a negative-control outcome, no vaccine status or influenza circulation interactions entered the model, demonstrating that influenza vaccination had no effect on the observed rate of UTI admissions. For each of our three outcomes of interest, a vaccine status and influenza A circulation interaction variable was included in the models, indicating an effect of vaccination. No vaccine status and influenza B interaction was found for pneumonia/influenza hospitalization or 30-day pneumonia/influenza deaths, indicating no evidence for a vaccine effect for influenza B-related outcomes. For influenza-associated all-cause deaths, the main effect of influenza B was negative (Table S2), suggesting that increasing circulation of influenza B was associated with lower outcome rates in the study population. Thus, it was not logical to estimate VE for prevention of influenza B-associated all-cause deaths.

During weeks when 5% of respiratory specimens tested positive for influenza A (weeks with moderate influenza activity), VE against influenza-associated all-cause deaths, 30-day pneumonia/influenza deaths, and pneumonia/influenza hospitalizations was 22% (95% CI, −6%–42%), 25% (95% CI, 13%–37%), and 19% (95% CI, 4%–31%), respectively (Table 2). VE was similar during weeks when 10% of specimens tested positive for influenza, a threshold often used to define peak weeks of activity. A plot of estimated VE by values of the proportion of specimens testing positive for influenza A from 0.01% to 25% is provided in the online supplement (Figure S2).

**Table 2:** Estimates of vaccine effectiveness (VE) for the prevention of influenza A-associated outcomes in community-dwelling Ontario residents aged ≥65 years (95% confidence interval [CI]) during weeks when 5% or 10% of respiratory specimens tested positive for influenza viruses.

Excess influenza-associated outcome	5% Circulation VE (95% CI)	10% Circulation VE (95% CI)
All-cause deaths	22% (−6, 42)	23% (−5, 42)
30-day pneumonia/influenza deaths	25% (13, 37)	26% (13, 38)
Pneumonia/influenza hospitalizations	19% (4, 31)	20% (6, 33)

**Figure 2:**
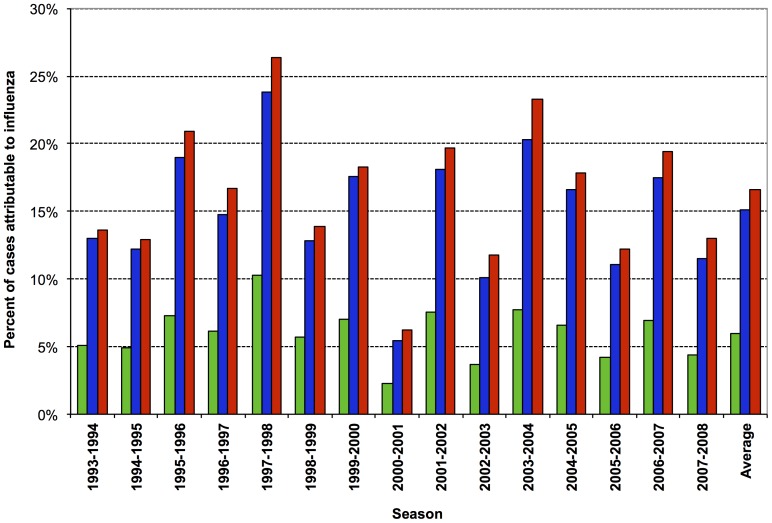
Proportion of outcomes that were influenza A-associated per season and overall. Percentage of all-cause mortality, 30-day pneumonia/influenza mortality, and pneumonia/influenza hospitalizations that were estimated to be influenza-associated during periods of influenza A circulation are indicated in the green, blue, and red columns, respectively.

A limited proportion of each outcome represented excess events occurring during weeks of influenza A circulation: a mean of 6.0% of all-cause deaths, 15.1% of 30-day pneumonia/influenza deaths, and 16.6% of pneumonia/influenza hospitalizations (Figure 2). These proportions varied considerably by season, as would be expected, given the considerable season-to-season differences in influenza circulation and relative intensity.

We estimated the annual numbers of each of the three outcomes potentially averted by vaccination with VE point estimates and predicted outcomes with or without a vaccine program (Table 3). During weeks when influenza was circulating, we estimated that influenza vaccination prevented 1.6% of all-cause deaths, 4.8% of 30-day pneumonia/influenza deaths, and 4.1% pneumonia/influenza hospitalizations among vaccinated individuals. Based on weekly vaccine coverage data, these results suggested that an average of 139 deaths (with a minimum of 43 in 1993/1994 and a maximum of 227 in 2004/2005) and 235 hospitalizations (with a minimum and maximum of 65 and 433 occurring in 2000/2001 and 2003/2004, respectively) were averted annually in Ontario by the influenza vaccination program. The numbers of elderly individuals needed to vaccinate to prevent one outcome were 5,124, 14,105, and 3,039 for all-cause deaths, 30-day pneumonia/influenza deaths, and pneumonia/influenza hospitalizations, respectively.

**Table 3:** Predicted number of cases averted by influenza vaccination, by study outcome.

Outcome	Season	No. weeks with influenza A detected	Cases among the vaccinated during weeks with influenza A detected	Cases averted by vaccination	% averted among vaccinees^a^
All-cause deaths					
	1993/1994	17	3636	43	1.2%
	1994/1995	25	5032	61	1.2%
	1995/1996	27	4126	77	1.8%
	1996/1997	28	5252	79	1.5%
	1997/1998	24	7359	202	2.7%
	1998/1999	39	9870	144	1.4%
	1999/2000	33	10519	207	1.9%
	2000/2001	26	7795	46	0.6%
	2001/2002	33	9116	182	2.0%
	2002/2003	36	12518	117	0.9%
	2003/2004	36	9183	197	2.1%
	2004/2005	41	12204	227	1.8%
	2005/2006	35	11867	136	1.1%
	2006/2007	35	11087	218	1.9%
	2007/2008	44	13311	156	1.2%
	Mean	32	8858	139	1.6%
30-day pneumonia/influenza deaths					
	1993/1994	17	398	16	3.9%
	1994/1995	25	526	20	3.7%
	1995/1996	27	406	25	5.8%
	1996/1997	28	656	30	4.4%
	1997/1998	24	934	78	7.7%
	1998/1999	39	1215	47	3.7%
	1999/2000	33	1368	86	5.9%
	2000/2001	26	837	14	1.6%
	2001/2002	33	972	60	5.8%
	2002/2003	36	1385	43	3.0%
	2003/2004	36	1159	89	7.1%
	2004/2005	41	1387	81	5.5%
	2005/2006	35	1210	42	3.4%
	2006/2007	35	1277	78	5.8%
	2007/2008	44	1447	51	3.4%
	Mean	32	1012	51	4.8%
Pneumonia/influenza hospitalizations					
	1993/1994	17	2227	70	3.0%
	1994/1995	25	2920	89	3.0%
	1995/1996	27	2092	108	4.9%
	1996/1997	28	3494	142	3.9%
	1997/1998	24	5140	357	6.5%
	1998/1999	39	6427	210	3.2%
	1999/2000	33	7525	397	5.0%
	2000/2001	26	4632	65	1.4%
	2001/2002	33	5532	281	4.8%
	2002/2003	36	7105	202	2.8%
	2003/2004	36	6454	433	6.3%
	2004/2005	41	8275	381	4.4%
	2005/2006	35	6695	196	2.8%
	2006/2007	35	6874	360	5.0%
	2007/2008	44	7824	237	2.9%
	Mean	32	5548	235	4.1%

a Percent averted among vaccinees is calculated as (cases averted / [total cases in the vaccinated population during weeks of influenza virus circulation + cases averted]) * 100

Linear models for all outcomes included interaction terms for the week of the year with vaccination status and mean temperature with vaccination status (Table S2). These terms controlled for time-varying biases within a season. Examination of the partial effects plots (not shown) for these factors show that substantial (upward) bias in VE would be found using traditional models during both the earlier part of the influenza season or for colder periods of the season (i.e. the pre- and post-influenza time periods). Thus our model, and specifically the aforementioned interaction terms, removed biases that resulted from observations taken during various periods of the year that have been observed in other vaccine effectiveness studies, such as that by Jackson *et al*. [10].

## Discussion

In this large population-based study, we applied a ‘ratio-of-ratios’ modeling approach to reduce the influence of difficult-to-measure individual-level confounders on the association between vaccination and three outcomes among older community-dwelling Ontario residents. The unmeasured confounders of greatest concern include physical frailty and dementia, which are incompletely captured in administrative health records and death certificates, but are likely associated with high mortality risks and low vaccination rates. During weeks of moderate-to-high influenza activity, influenza vaccination was associated with a (non-significant) 22% reduction in influenza-associated deaths (i.e. those deaths in the vaccinated population that would exceed an expected value in the absence of influenza circulation). Excess deaths occurring within 30 days of a pneumonia/influenza hospitalization, and excess pneumonia/influenza hospitalizations were significantly reduced, by 25% and 19%, respectively. As expected, no benefit from influenza vaccination was observed for UTI hospitalizations. Despite demonstrating a moderate level of VE for the three primary outcomes, the predicted mean annual numbers of events prevented in Ontario were small (139 all-cause deaths, 51 deaths in the 30 days following a pneumonia/influenza hospitalization, and 235 pneumonia/influenza hospitalizations) because the proportions of these deaths and hospitalizations that were associated with influenza activity were small. It is important to highlight that the protective effects of large-scale vaccination campaigns might be greater than these estimates because of indirect protective effects of such campaigns (i.e., herd-immunity) [34], [35]. However, the quantification of indirect effects is extremely difficult for diseases that cannot be definitely diagnosed without specific laboratory testing, including influenza infections [36]. Thus, we have focused on the direct and more conservative benefits of influenza vaccination in this study.

We estimated the effectiveness of influenza vaccination for prevention of outcomes that: 1) occurred during weeks when specific laboratory testing for influenza revealed that influenza viruses were circulating at pre-specified levels in Ontario; and 2) were above a seasonally adjusted baseline of counts for each outcome. These outcomes are in contrast to those used in many other cohort studies, in which all events occurring during periods of any influenza virus circulation were used as outcomes measures. The importance of this difference is easily demonstrated: because only 6.0% of all deaths among Ontarians aged ≥65 years occurred during weeks when influenza circulated were above a seasonal baseline (and thus were categorized as influenza-associated), a VE of 22% for this outcome represented a 1.6% reduction in deaths among vaccinated individuals. This 1.6% reduction can be interpreted as a population-based estimate of vaccine effects on all deaths occurring during periods of influenza virus circulation. A meta-analysis of cohort studies calculated a VE of 47% for the prevention of all-cause mortality among community dwelling elderly during influenza seasons [8]. Studies by Jackson *et al*. and Mangtani *et al*., demonstrated the bias inherent in cohort analyses by detecting putative vaccine benefits for non-specific outcomes when influenza activity is nil [10], [16]. Thus, we confirm with data from the most populous province in Canada that VE estimates not accounting for individual-level baseline risks for mortality are unrealistically optimistic. Because greater proportions of 30-day pneumonia/influenza deaths and pneumonia/influenza hospitalizations were attributed to influenza, greater percentages of these more specific events were averted by influenza vaccination, and thus the VE estimates of 25% and 19%, respectively, against these outcomes are likely more robust.

Our results can be compared directly with those from a few other studies that explicitly sought to adjust for unmeasured confounding, including healthy vaccinee effects. Using medical chart review to collect data on covariates not traditionally available in administrative data, Jackson *et al*. estimated a VE of 8% (95% CI, −10%–23%) against community-acquired pneumonia during influenza seasons [17], consistent with our estimate of 19% VE (CI 4%–31%) and a 4.1% reduction of all pneumonia/influenza hospitalizations occurring during periods of influenza virus circulation among vaccinated individuals. The Armstrong *et al*. study estimate of VE for influenza-associated all-cause mortality was 85% during 1996–2000. The cohort size of ∼25,000 in that study meant that its 95% CI of 13%–100% covered essentially the entire possible range of vaccine benefits; thus their CI does include our point estimate [18], [19]. Fireman *et al*. estimated that influenza vaccination was associated with a 47% reduction (95% CI not provided) in influenza-associated (as opposed to all) deaths between 1996 and 2005 in a single U.S. managed care plan [20]. The same group also reported a 28% reduction (95% CI not provided) in influenza-associated pneumonia/influenza hospitalizations [37], which is similar to our VE estimate for pneumonia/influenza hospitalizations. Another recent study used an instrumental variable approach to estimate influenza VE. The use of an instrumental variable to adjust for unmeasured confounding is common in econometric analyses of observational data [38], [39]. In that Ontario study conducted during the 2000-2009 influenza seasons, VE among adults aged ≥65 years was 6% (95% CI, 0%–16%) for all-cause deaths and 14% (95% CI, 8%–21%) for a composite outcome of a pneumonia/influenza hospitalization or death [40]; these results are comparable to our results on the reduction in deaths due to all-cause deaths or pneumonia/influenza hospitalization. The instrumental variable method depends on finding a covariate closely associated with vaccination, but unrelated to outcome; it is often difficult to find a good instrument. Hence, an advantage of the regression methods we used is that they are more broadly applicable and likely more generalizable for use in other populations.

This study has a number of strengths. We studied influenza vaccine effectiveness during 15 influenza seasons, representing ∼21 million person-years of observation. We included temperature in our analyses, a variable that both affects winter mortality among older persons and is associated with influenza circulation, in temperate regions [31], [41], which may have improved the precision of our VE estimates. By further developing methods pioneered by Armstrong *et al*., and using three outcomes of varying specificity, we estimated the effectiveness of influenza vaccination in preventing serious influenza-associated events in individuals aged ≥65 years to a greater level of precision than previously. We also carefully specified whether a specific VE estimate applied to all events occurring during an influenza season, or to excess events occurring during periods of a specific level of influenza activity (e.g., when 5% of specimens submitted for influenza testing were positive). Finally, our analyses use a generalized linear model framework, and therefore are easy to implement using standard statistical analysis software packages.

Our study also has a number of limitations. First, although most Ontario residents aged ≥65 years receive influenza vaccination in physician offices, some are vaccinated in settings where billing claims are not submitted (e.g., clinics organized by public health departments). Billing claims were found to be 75% sensitive and 90% specific compared with self-report of influenza vaccination in one study [23]. Misclassification resulting from use of billing data would bias our results towards the null as the unvaccinated group's risk would be falsely lowered because of the inclusion of misclassified vaccinated individuals. Second, cause-specific mortality data were not available. We used excess mortality within the 30 days following a pneumonia/influenza hospitalization to provide an outcome more specific for influenza than all-cause deaths. Third, measures of influenza virus circulation are a key data element in our analyses and the influenza surveillance data we used were potentially susceptible to ascertainment and testing biases over time. However, there were no major changes in data collection or laboratory methods during the study, and the weekly proportion of tests positive for influenza is a robust measure of viral activity [32]. Fourth, because only a small number (2%) of influenza tests were positive for influenza B viruses, we could not provide a specific estimate of VE for influenza B-associated events, so VE estimates could be made only for influenza A-related outcomes. Finally, in common with all population-based retrospective cohort studies, we did not have data on laboratory-confirmed influenza infections from a per-protocol prospective testing scheme. It is unlikely that such data will ever be collected on a community- or province-wide scale because of the obvious logistical and resource requirements.

Based on our results and those from other studies, influenza vaccines that are more effective in preventing serious complications of influenza infections are clearly needed, particularly for older persons. Several strategies offering potentially more effective vaccines are being pursued. For example, a high-dose inactivated vaccine was licensed recently in the United States based on superior immunogenicity data [42]. It will be important to assess whether new influenza vaccines prevent more serious albeit rare complications of influenza infections than the decades-old standard inactivated vaccines. Large observational studies using bias-reducing methods likely represent the only possible option to study the relative effectiveness of new versus standard influenza vaccines for the serious outcomes of greatest interest, including mortality. In addition, the methods we used may also be suitable for evaluating other large-scale public health interventions in populations in which unmeasured individual-level characteristics like frailty and dementia, for example, may be important confounders.

## Supporting Information

Figure S1 **Weekly physician billing claims submitted with influenza-specific and generic vaccination codes.** Physician billing claims for generic vaccination codes are represented by gray bars and influenza-specific vaccination codes are represented by black bars. The bars are stacked. Prior to the introduction of influenza-specific codes in 1998, physicians used generic codes when billing for influenza vaccination, which are evident as substantial spikes above a fixed baseline. There was a gradual increase in the use of the influenza-specific codes, and a corresponding gradual reduction in the use of the generic codes. We estimated that only 4% of the combined influenza-specific and generic vaccination claims during weeks of the annual influenza vaccination campaigns are not for influenza vaccination.
(TIFF)

Figure S2 **Change in vaccine effectiveness as a function of circulating influenza levels.** Vaccine effectiveness is a continuous function dependent on the level of influenza circulation within the population. Black lines represent the expected VE at any level of circulation and the dashed red lines indicate the upper and lower 95% confidence bands. Note that VE changes relatively little across circulation values and in a linear manner.
(TIFF)

Table S1 **Variables potentially included in the log-linear regression model.**
(DOCX)

Table S2 **Enumeration of all predictors included in the final log-linear regression models for each outcome.**
(DOCX)

Table S3 **Number of individuals, vaccinations and outcomes for each study year, stratified by age group (65–74 years and ≥75 years).**
(DOCX)
