# Supplementary figures and images for: Effectiveness of Inactivated Influenza Vaccines in Preventing Influenza-Associated Deaths and Hospitalizations among Ontario Residents Aged ≥65 Years: Estimates with Generalized Linear Models Accounting for Healthy Vaccinee Effects
Source: PLoS One. 2013 Oct 16;8(10):e76318. doi: 10.1371/journal.pone.0076318 (PMC3797825; doi:10.1371/journal.pone.0076318)

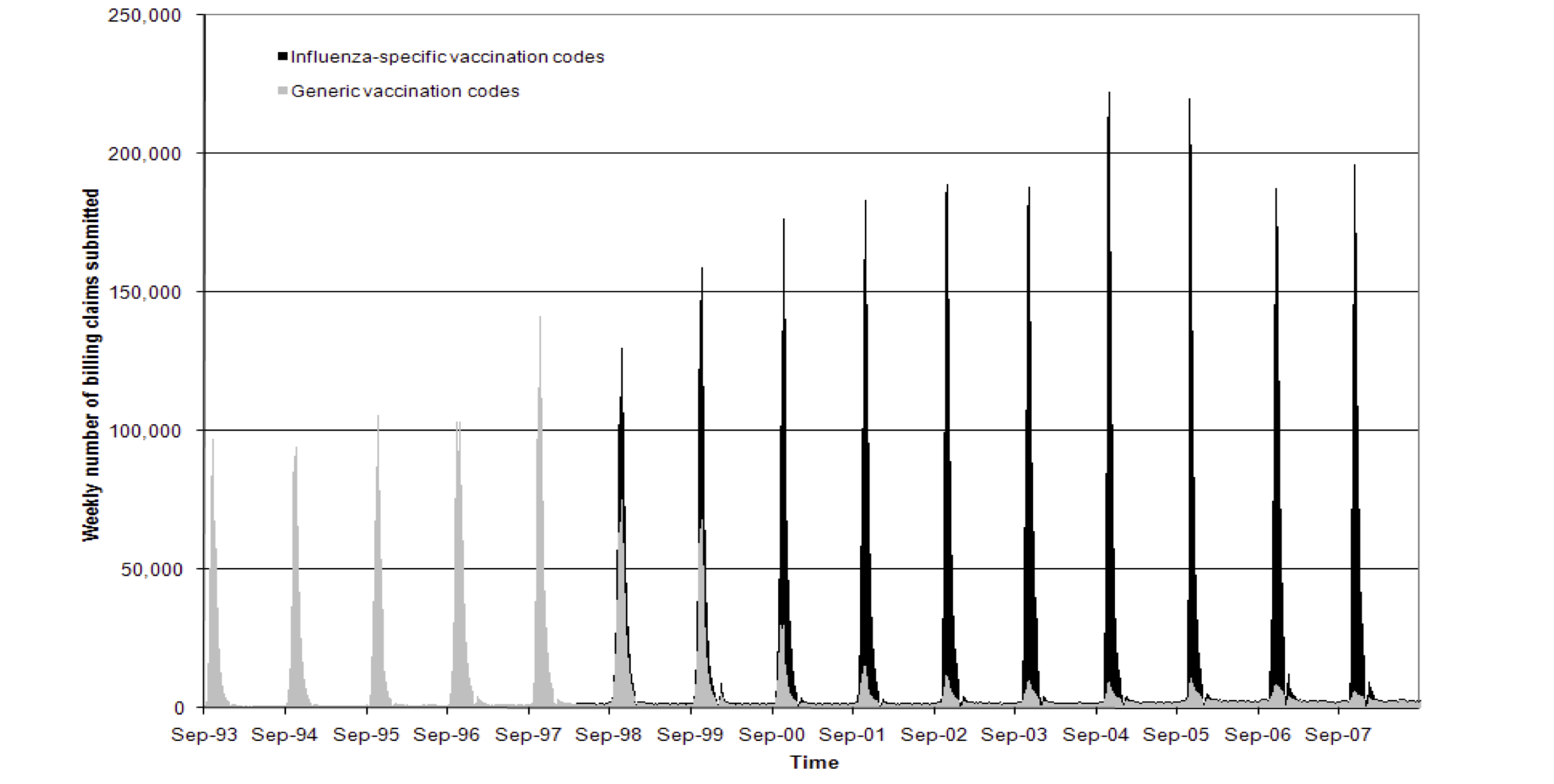

Supplement: Figure S1 — Weekly physician billing claims submitted with influenza-specific and generic vaccination codes. Physician billing claims for generic vaccination codes are represented by gray bars and influenza-specific vaccination codes are represented by black bars. The bars are stacked. Prior to the introduction of influenza-specific codes in 1998, physicians used generic codes when billing for influenza vaccination, which are evident as substantial spikes above a fixed baseline. There was a gradual increase in the use of the influenza-specific codes, and a corresponding gradual reduction in the use of the generic codes. We estimated that only 4% of the combined influenza-specific and generic vaccination claims during weeks of the annual influenza vaccination campaigns are not for influenza vaccination. (TIFF) [file pone.0076318.s001.tiff]

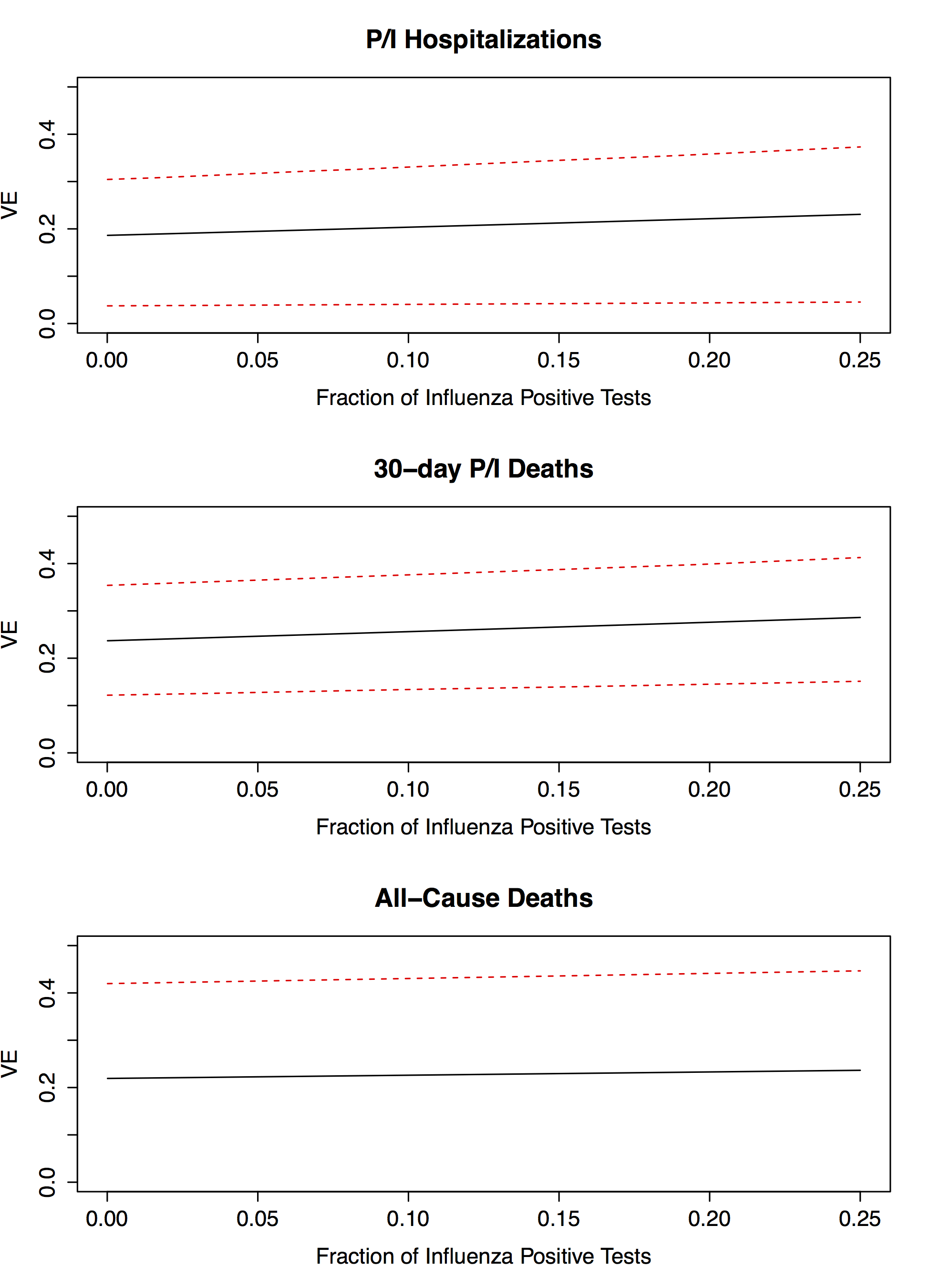

Supplement: Figure S2 — Change in vaccine effectiveness as a function of circulating influenza levels. Vaccine effectiveness is a continuous function dependent on the level of influenza circulation within the population. Black lines represent the expected VE at any level of circulation and the dashed red lines indicate the upper and lower 95% confidence bands. Note that VE changes relatively little across circulation values and in a linear manner. (TIFF) [file pone.0076318.s002.tiff]
