# Supplementary material for: Effectiveness of Inactivated Influenza Vaccines in Preventing Influenza-Associated Deaths and Hospitalizations among Ontario Residents Aged ≥65 Years: Estimates with Generalized Linear Models Accounting for Healthy Vaccinee Effects
Source: PLoS One. 2013 Oct 16;8(10):e76318. doi: 10.1371/journal.pone.0076318 (PMC3797825; doi:10.1371/journal.pone.0076318)
Supplement: Table S1 — Variables potentially included in the log-linear regression model. (DOCX) [file pone.0076318.s003.docx]

**Table S1. Variables potentially included in the log-linear regression model.**

| **Predictor** | **Type** | **Purpose** |
| --- | --- | --- |
| Vaccination status^1^ | Factor | Primary exposure |
| Influenza A viral circulation^1^ | Continuous, Percentage | Estimate the effect of influenza A viruses on an outcomes |
| Influenza B viral circulation^1^ | Continuous, Percentage | Estimate the effect of influenza B viruses on an outcomes |
| Influenza A viral circulation x vaccination status^1^ | Interaction | Estimate the protective effect of vaccination against influenza A |
| Influenza B viral circulation x vaccination Status^1^ | Interaction | Estimate the protective effect of vaccination against influenza B |
| Week of the year (1…52) | Natural Spline | Account for seasonal changes in the basal outcome rate |
| Week of the year x vaccination status | Interaction | Account for reduced vaccine benefits of doses given late in a season |
| Week of the study (1…783) | Natural Spline | Account for long term (slow moving) changes in outcome rates |
| Week of the study x vaccination status | Interaction | Account for any long term, time varying effects of vaccination |
| Mean temperature | Natural Spline | Account for increases / decreases in outcomes due to inclement weather |
| Mean temperature x vaccination status | Interaction | Account for any possible effect of vaccination that co-vary with temperature |
| Sex | Factor | Correct for any bias in outcome due to the sex of a vaccinee |
| Sex x vaccination status | Interaction | Correct for vaccine bias that also depends on the sex of the recipient |
| Universal influenza immunization program (UIIP) | Factor | Correct for any bias in outcome caused by a change in the Ontario vaccination plan |
| UIIP x vaccination status | Interaction | Account for difference between vaccination groups pre- and post-UIIP introduction |
| Offset^1^ | Continuous | Person-days of observation in a particular stratum (e.g. sex, age group, and vaccination status) for a given week during the study period |

^1^Term included in the baseline regression model that was not subject to our inclusion or exclusion criterion for entry or removal (respectively) from the final model. See text for details on the model choice procedure.
