# Supplementary material for: Effectiveness of Inactivated Influenza Vaccines in Preventing Influenza-Associated Deaths and Hospitalizations among Ontario Residents Aged ≥65 Years: Estimates with Generalized Linear Models Accounting for Healthy Vaccinee Effects
Source: PLoS One. 2013 Oct 16;8(10):e76318. doi: 10.1371/journal.pone.0076318 (PMC3797825; doi:10.1371/journal.pone.0076318)
Supplement: Table S2 — Enumeration of all predictors included in the final log-linear regression models for each outcome. (DOCX) [file pone.0076318.s004.docx]

**Table S2. Enumeration of all predictors included in the final log-linear regression models for each outcome.**

|  | **All-cause deaths model** | | **30-day P/I deaths model** | | **P/I hospitalization model** | | **UTI model** | | |  |
| --- | --- | --- | --- | --- | --- | --- | --- | --- | --- | --- |
| **Predictor** | **Parameter estimate** | **P-value** | **Parameter estimate** | **P-value** | **Parameter estimate** | **P-value** | **Parameter estimate** | **P-value** | |  |
| (Intercept) | -10.1131 (0) | <0.0001 | -12.8854 (0) | <0.0001 | -10.9693 (0) | <0.0001 | -10.7230 (0) | | <0.0001 | |
| Vaccination status (Vaccinated) | -0.5272 (3) | <0.0001 | 0.1491 (6) | 0.0692 | 0.2080 (8) | 0.0006 | 0.0432 (3) | | 0.1748 | |
| Influenza A circulation | 0.7901 (7) | <0.0001 | 2.0916 (3) | <0.0001 | 2.2258 (2) | <0.0001 |  | |  | |
| Influenza B circulation | -0.4167 (8) | 0.0104 |  |  | 0.5398 (7) | 0.0893 | 0.0225 (7) | | 0.9364 | |
| Age group (75+) | 1.2151 (1) | <0.0001 | 1.4664 (1) | <0.0001 | 1.1439 (1) | <0.0001 | 1.1727 (1) | | <0.0001 | |
| Sex (Male) | 0.4147 (2) | <0.0001 | 0.6346 (2) | <0.0001 | 0.4511 (3) | <0.0001 | -0.2774 (2) | | <0.0001 | |
| UIIP | 0.0486 (9) | 0.0034 | -0.0420 (8) | 0.1992 |  |  | 0.0244 (8) | | 0.2975 | |
| Influenza A x Vaccination status interaction | -0.1732 (15) | 0.0839 | -0.4955 (10) | 0.0002 | -0.4147 (10) | 0.0156 |  | |  | |
| Influenza B x Vaccination status interaction | 0.3905 (16) | 0.0429 |  |  |  |  |  | |  | |
| Vaccination status x Age group interaction | -0.1126 (11) | <0.0001 | -0.1177 (9) | <0.0001 | -0.1427 (9) | <0.0001 | -0.1204 (9) | | <0.0001 | |
| Vaccination status x Sex interaction | 0.0683 (12) | <0.0001 |  |  |  |  |  | |  | |
| Vaccination status x UIIP | -0.0740 (14) | <0.0001 |  |  |  |  |  | |  | |
| Natural cubic spline for week of the study period |  |  |  |  |  |  |  | |  | |
| Segment 1 | -0.4429 (4) | <0.0001 | -0.2937 (4) | <0.0001 | -0.3454 (4) | <0.0001 | 0.0912 (4) | | 0.0378 | |
| Segment 2 |  |  |  |  |  |  |  | |  | |
| Segment 3 |  |  |  |  |  |  |  | |  | |
| Segment 4 |  |  |  |  |  |  |  | |  | |
| Segment 5 |  |  |  |  |  |  |  | |  | |
| Segment 6 |  |  |  |  |  |  |  | |  | |
| Natural cubic spline for week of the study year |  |  |  |  |  |  |  | |  | |
| Segment 1 | 0.9012 (6) | <0.0001 | 0.3065 (5) | 0.0001 | 0.2689 (5) | 0.0004 | -0.1631 (6) | | 0.0007 | |
| Segment 2 | -0.1971 (6) | <0.0001 | -0.4074 (5) | <0.0001 | -0.5061 (5) | <0.0001 | -0.0194 (6) | | 0.3067 | |
| Segment 3 |  |  |  |  |  |  |  | |  | |
| Segment 4 |  |  |  |  |  |  |  | |  | |
| Segment 5 |  |  |  |  |  |  |  | |  | |
| Segment 6 |  |  |  |  |  |  |  | |  | |
| Natural cubic spline for temperature |  |  |  |  |  |  |  | |  | |
| Segment 1 | -0.3433 (5) | <0.0001 | 0.1280 (7) | 0.1884 | 0.0690 (6) | 0.4432 | 0.0771 (5) | | 0.1213 | |
| Segment 2 |  |  | -0.1424 (7) | 0.0059 | -0.1436 (6) | 0.0112 |  | |  | |
| Segment 3 |  |  |  |  |  |  |  | |  | |
| Segment 4 |  |  |  |  |  |  |  | |  | |
| Segment 5 |  |  |  |  |  |  |  | |  | |
| Segment 6 |  |  |  |  |  |  |  | |  | |
| Vaccination status x natural cubic spline of week of the study period interaction |  |  |  |  |  |  |  | |  | |
| Segment 1 |  |  | -0.1561 (11) | <0.0001 | -0.1008 (11) | 0.0009 | -0.0422 (11) | | 0.0374 | |
| Segment 2 |  |  |  |  |  |  |  | |  | |
| Segment 3 |  |  |  |  |  |  |  | |  | |
| Segment 4 |  |  |  |  |  |  |  | |  | |
| Segment 5 |  |  |  |  |  |  |  | |  | |
| Segment 6 |  |  |  |  |  |  |  | |  | |
| Vaccination status x natural cubic spline of week of the study year interaction |  |  |  |  |  |  |  | |  | |
| Segment 1 | 0.4210 (10) | <0.0001 | -0.5715 (12) | 0.0279 | -0.8406 (12) | 0.0003 | 0.0067 (10) | | 0.9176 | |
| Segment 2 | 0.4380 (10) | <0.0001 | 0.2218 (12) | <0.0001 | 0.1918 (12) | <0.0001 | 0.1276 (10) | | <0.0001 | |
| Segment 3 |  |  |  |  |  |  |  | |  | |
| Segment 4 |  |  |  |  |  |  |  | |  | |
| Segment 5 |  |  |  |  |  |  |  | |  | |
| Segment 6 |  |  |  |  |  |  |  | |  | |
| Vaccination status x natural cubic spline for temperature |  |  |  |  |  |  |  | |  | |
| Segment 1 | 0.2415 (13) | 0.0004 | 0.3666 (13) | 0.1011 | 0.7174 (13) | 0.0003 |  | |  | |
| Segment 2 |  |  | 0.2909 (13) | 0.0019 | 0.3701 (13) | <0.0001 |  | |  | |
| Segment 3 |  |  |  |  |  |  |  | |  | |
| Segment 4 |  |  |  |  |  |  |  | |  | |
| Segment 5 |  |  |  |  |  |  |  | |  | |
| Segment 6 |  |  |  |  |  |  |  | |  | |

The deviance ratios (similar to R^2^) for the models having all-cause deaths, 30-day pneumonia/influenza deaths, pneumonia/influenza hospitalizations, and urinary tract infections as outcomes were 96.3%, 84.2%, 89.7%, and 92.6% respectively. The number in parentheses next to the slope estimate for each independent variable is the order of inclusion into the model. Other than the default intercept of the models (all numbered 0), order of inclusion into the model represents the relative importance for explaining deviance. For example, in every model age group was the first variable included, indicating that age was the most important factor in explaining any of the three outcomes.
