# Supplementary material for: Effectiveness of Inactivated Influenza Vaccines in Preventing Influenza-Associated Deaths and Hospitalizations among Ontario Residents Aged ≥65 Years: Estimates with Generalized Linear Models Accounting for Healthy Vaccinee Effects
Source: PLoS One. 2013 Oct 16;8(10):e76318. doi: 10.1371/journal.pone.0076318 (PMC3797825; doi:10.1371/journal.pone.0076318)
Supplement: Table S3 — Number of individuals, vaccinations and outcomes for each study year, stratified by age group (65–74 years and ≥75 years). (DOCX) [file pone.0076318.s005.docx]

| **Study Year** | **Age Group (Years)** | **N** | **Influenza vaccinations (%)** | **All-cause deaths** | | **30-day P&I deaths** | | **P&I hospitalizations** | |
| --- | --- | --- | --- | --- | --- | --- | --- | --- | --- |
|  |  |  |  | **Vaccinated** | **Unvaccinated** | **Vaccinated** | **Unvaccinated** | **Vaccinated** | **Unvaccinated** |
| 1993/1994 | 65-74 | 761,276 | 266,794 (35.0) | 4,490 | 12,365 | 348 | 851 | 2,345 | 5,079 |
|  | ≥75 | 452,802 | 170,726 (37.7) | 7,924 | 20,900 | 839 | 1,793 | 3,951 | 7,992 |
| 1994/1995 | 65-74 | 782,787 | 284,913 (36.4) | 4,619 | 12,488 | 374 | 939 | 2,519 | 5,366 |
|  | ≥75 | 464,870 | 186,170 (40.0) | 8,538 | 21,277 | 933 | 1,852 | 4,429 | 8,435 |
| 1995/1996 | 65-74 | 791,865 | 300,748 (38.0) | 4,911 | 12,091 | 386 | 885 | 2,541 | 4,981 |
|  | ≥75 | 487,675 | 205,119 (42.1) | 9,333 | 21,510 | 948 | 1,860 | 4,362 | 7,993 |
| 1996/1997 | 65-74 | 800,861 | 326,696 (40.8) | 5,262 | 11,473 | 431 | 873 | 2,988 | 4,982 |
|  | ≥75 | 507,304 | 234,870 (46.3) | 10,550 | 20,844 | 1,204 | 2,049 | 5,381 | 8,405 |
| 1997/1998 | 65-74 | 805,690 | 349,682 (43.4) | 5,328 | 10,956 | 483 | 879 | 3,306 | 4,706 |
|  | ≥75 | 528,472 | 259,757 (49.2) | 11,286 | 20,668 | 1,334 | 2,096 | 6,228 | 8,463 |
| 1998/1999 | 65-74 | 810,409 | 356,634 (44.0) | 5,398 | 10,659 | 504 | 902 | 3,397 | 4,997 |
|  | ≥75 | 549,505 | 279,024 (50.8) | 11,817 | 20,384 | 1,434 | 2,036 | 6,598 | 8,838 |
| 1999/2000 | 65-74 | 812,306 | 389,412 (47.9) | 5,656 | 10,034 | 555 | 814 | 3,685 | 5,201 |
|  | ≥75 | 570,413 | 309,854 (54.3) | 12,891 | 19,615 | 1,573 | 2,077 | 7,499 | 9,523 |
| 2000/2001 | 65-74 | 816,160 | 467,631 (57.3) | 6,352 | 8,868 | 568 | 680 | 3,875 | 4,145 |
|  | ≥75 | 592,382 | 366,976 (61.9) | 14,501 | 18,472 | 1,556 | 1,706 | 7,865 | 7,701 |
| 2001/2002 | 65-74 | 820,946 | 458,434 (55.8) | 6,092 | 9,005 | 559 | 693 | 3,719 | 4,147 |
|  | ≥75 | 613,963 | 379,397 (61.8) | 14,442 | 18,787 | 1,615 | 1,845 | 8,296 | 8,438 |
| 2002/2003 | 65-74 | 826,859 | 434,769 (52.6) | 5,705 | 9,162 | 488 | 639 | 3,123 | 3,882 |
|  | ≥75 | 634,690 | 379,506 (59.8) | 14,023 | 20,061 | 1,479 | 1,878 | 6,980 | 8,107 |
| 2003/2004 | 65-74 | 834,591 | 470,946 (56.4) | 5,795 | 8,566 | 518 | 642 | 3,510 | 3,858 |
|  | ≥75 | 653,933 | 409,068 (62.6) | 14,833 | 19,861 | 1,672 | 1,997 | 8,278 | 8,435 |
| 2004/2005 | 65-74 | 845,764 | 465,525 (55.0) | 5,653 | 8,489 | 474 | 649 | 3,532 | 3,910 |
|  | ≥75 | 670,647 | 416,809 (62.2) | 15,230 | 19,911 | 1,682 | 1,924 | 8,652 | 8,531 |
| 2005/2006 | 65-74 | 853,706 | 456,931 (53.5) | 5,082 | 8,385 | 431 | 557 | 2,885 | 3,561 |
|  | ≥75 | 689,961 | 421,664 (61.1) | 14,350 | 20,150 | 1,477 | 1,839 | 7,428 | 8,128 |
| 2006/2007 | 65-74 | 868,188 | 430,899 (49.6) | 4,513 | 9,099 | 421 | 681 | 2,651 | 4,259 |
|  | ≥75 | 712,695 | 415,945 (58.4) | 13,463 | 22,819 | 1,419 | 2,190 | 7,098 | 9,636 |
| 2007/2008 | 65-74 | 887,526 | 415,233 (46.8) | 4,288 | 9,365 | 341 | 677 | 2,596 | 4,200 |
|  | ≥75 | 732,673 | 410,010 (56.0) | 13,653 | 23,798 | 1,495 | 2,243 | 7,253 | 9,719 |
| Average | 65-74 | 821,262 | 391,683 (47.7) | 5,276 | 10,067 | 459 | 757 | 3,111 | 4,485 |
|  | ≥75 | 590,799 | 322,993 (54.7) | 12,456 | 20,604 | 1,377 | 1,959 | 6,687 | 8,556 |

**Table S3. Number of individuals, vaccinations and outcomes for each study year, stratified by age group (65-74 years and ≥75 years)**
